# The Tonic Treatment of Syphilis

**Published:** 1877-03

**Authors:** 


THE TONIC TREATMENT OF SYPHILIS. By E. L. Keyes, A.M., M.D., Adjunct Professor of Surgery and Professor of Dermatology in the Bellevue Hospital Medical College; Surgeon to the Bellevue Hospital; Consulting Surgeon to the Charity Hospital; Consulting Dermatologist to the Bellevue Bureau of Out-door Relief, etc. New York: D. Appleton & Co., Publishers. 1877.

The popular idea of the destructive tendency of mercury is not confined to the unprofessional. Many in the profession are far from favoring its constant and continued administration in any dose.

The stand point from which the physician reasons is founded on the fact of its defibrinating effect and the general impoverishment and debility that ensues upon its continued use. From this emanates the popular idea, and, actuated by ignorance, seeks a characteristic extreme. The isolated administration of the drug, in any dose in health or disease, is attended by lasting injury, in the minds of many of the people. This idea is exploded by the experiments of the author, which demonstrates that the alterative action of mercury is really tonic in small doses, as it increases the red corpuscles of the blood. Through experiments made in a number of cases, by the aid of the hæmatimetre, the following conclusions have been deduced:

1. "Mercury decreases the number of red cells when given in excess. * * *

2. Syphilis diminishes the number of red corpuscles below the healthy standard.

3. Mercury in small doses, continued for a short or long period in syphilis, alone or with iodide of potassium, increases the number of red corpulcles in the blood, and maintains a high standard of the same.

4. Mercury in small doses acts as a tonic upon healthy animals, increasing their weight. In larger doses it is debilitating.

5. Mercury in small doses is a tonic (for a time, at least as long as the experiments lasted) to individuals in fair health, not syphilitic. On such individuals, it increases the number of the red corpuscles."

By this it seems that the administration of small doses is the desideratum to success. In fact, so minute are they that the most scrupulous homœopothist could not object to them; as, for instance, the one-hundreth of a grain of blue mass at a dose!

The same result is claimed for iodide of potassium as to the tonic effect, except that in syphilis this effect is greater from the iodide. But then the iodide seems not to modify or delay the symptoms as the mercury does. With this view the mercury is commenced as soon as the diagnosis is made, and administered continuously for the term of two years.

The objects of the work, as expressed by the author, is to show that : 1. "Mercury is generally recognized as capable of overcoming the symptoms of syphilis, and postponing their appearance : 2. Mercury in minute doses, long continued, is tonic." Should this theory be established by the chemical experience of the profession generally, occasion will be furnished to illustrate the conservative and liberal views of the fraternity, by adopting this homœopathic measure.

We have long looked upon mercury as our best remedy in the treatment of syphilis, but we are free to confess that we would have but small faith in so small a dose as the one-hundreth of a grain of blue mass. We would have to be thoroughly impressed before we could adopt this infinitesimalism. This is our principal objection to the work. Many points of interest are found in it, and those feeling a concern in the subject would do well to give it a thorough perusal.

